# Editorial

**Published:** 1838-03-14

**Authors:** 


### MEDICAL EXAMINER.

PHILADELPHIA.

Wednesday, March 14, 1838.

We insert the following note from Dr. Harlan, in which he simply expresses his unwillingness to pursue the controversy with Professor Gibson, through the medium of our journal. Here, as far as we are concerned, the matter very properly ends.

Philadelphia, February 28th, 1838.

*To the Editors of the Medical Examiner.*

Gentlemen:

In the fifth number of your journal, just received, I find appended to your report of my lecture, delivered at the Philadelphia Hospital, on the 3d inst. some remarks of Dr. Gibson's, which I consider of too personal a nature. My animadversions on Dr. G.'s lecture were confined, as much as possible, to the operation, and not extended to the operator. My name having been mentioned to the class by Dr. G., as opposed to the operation, and my reasons having been withheld, it became my duty, on this account alone, to communicate the grounds of my opinion to the class—independently of the important principle, involved in the discussion.

I shall, of course, not desire the use of your journal, for the purpose of answering Dr. G.'s personalities, but intend, in a few days, to take occasion to set the matter right, in the eyes of the public, in a manner, I trust, to his perfect conviction, if not to his entire satisfaction.

I have the honor to be, very respectfully,

Your obedient servant,

R. Harlan.

---

